# Fine‐scale frequency differentiation along a herbivory gradient in the trichome dimorphism of a wild *Arabidopsis*

**DOI:** 10.1002/ece3.2830

**Published:** 2017-02-28

**Authors:** Yasuhiro Sato, Hiroshi Kudoh

**Affiliations:** ^1^ Center for Ecological ResearchKyoto UniversityOtsuShigaJapan; ^2^ Present address: Department of Plant Life SciencesFaculty of AgricultureRyukoku UniversityYokotani 1‐5, Seta Oe‐choOtsuShiga520‐2194Japan

**Keywords:** *Arabidopsis halleri*, ecogeographic differentiation, plant–herbivore interaction, polymorphism, trichome

## Abstract

Geographic variation is commonly observed in plant resistance traits, where plant species might experience different selection pressure across a heterogeneous landscape. *Arabidopsis halleri* subsp. *gemmifera* is dimorphic for trichome production, generating two morphs, trichome‐producing (hairy) and trichomeless (glabrous) plants. Trichomes of *A. halleri* are known to confer resistance against the white butterfly, cabbage sawfly, and brassica leaf beetle, but not against flea beetles. We combined leaf damage, microclimate, and microsatellite loci data of 26 *A. halleri* populations in central Japan, to explore factors responsible for fine‐scale geographic variation in the morph frequency. We found that hairy plants were less damaged than glabrous plants within populations, but the among‐site variation was the most significant source of variation in the individual‐level damage. Fixation index ($G_{\mathrm{st}}^{\prime \prime }$) of a putative trichome locus exhibited a significant divergence along population‐level damage with an exception of an outlier population, inferring the local adaptation to herbivory. Notably, this outlier was a population wherein our previous study reported a balancing role of the brassica leaf beetle *Phaedon brassicae* on the morph frequency. This differentiation of the trichome locus was unrelated to neutral genetic differentiation (evaluated by $G_{\mathrm{st}}^{\prime \prime }$ of microsatellite loci) and meteorological factors (including temperature and solar radiation). The present findings, combined with those of our previous work, provide suggestive evidence that herbivore‐driven divergence and occasional outbreak of a specific herbivore have jointly contributed to the ecogeographic pattern in the frequency of two morphs.

## 1 Introduction

How species interactions shape phenotypic variation in a heterogeneous landscape is a central question in ecology and evolutionary biology (Farkas, Hendry, Nosil, & Beckerman, 2015; Thompson, 2005; Urban et al., 2008). As a major group of sessile organisms, many plant species develop resistance traits against herbivores (Fritz & Simms, 1992; Schoonhoven, van Loon, & Dicke, 2005). In the concept of geographic mosaic of coevolution (Thompson, 1999, 2005), plant–herbivore interactions and selection pressure vary across a heterogeneous landscape, driving local adaptation of a plant species to prevalent herbivore regimes (Bernhardsson et al., 2013; Sakata, Yamasaki, Isagi, & Ohgushi, 2014; Züst et al., 2012). Because resistance traits are often costly for plant growth (Koricheva, 2002; Mauricio, 1998; Sletvold, Huttunen, Handley, Kärkkäinen, & Ågren, 2010), an optimal defense level would depend on a trade‐off between costs and benefits of resistance. Provided that the intrinsic cost is constant within a species, the increasing risk of herbivory should be positively related to the benefit of resistance and thus predict the evolution of higher resistance levels (e.g., Løe, Toräng, Gaudeul, & Ågren, 2007; Sakata et al., 2014; Züst et al., 2012).

In host–parasite interactions, complex geographic variation in a host resistance polymorphism may be shaped by balancing and divergent selection (Thompson, 1999, 2005). In a plant–herbivore system, for example, Berenbaum and Zangerl (1998) have suggested that selective herbivory by a parsnip webworm on abundant chemotypes of the wild parsnip *Pastinaca sativa* results in negative frequency‐dependent selection and consequently maintains multiple chemotypes within a population. In contrast, among populations, host alternation of the webworm to another parsnip species provided *P. sativa* with hot and cold spots of coevolution that drive divergent selection of *P. sativa* resistance to the webworm (Zangerl & Berenbaum, 2003). This selection mosaic occurs because spatial distribution of host plant species for the webworm varies among populations. These lines of evidence from the wild parsnip suggest that negative frequency‐dependent selection within a population and divergent selection among populations counteract each other, shaping complex geographic variations in antiherbivore resistance. However, ecological drivers of the geographic mosaic of plant resistance remain poorly understood.

It is noteworthy here that, when interspecific interactions are spatially structured, stochastic gene flow might connect adjacent populations and thereby contribute to the pattern of geographic variation in phenotypic traits (Farkas et al., 2015; Thompson, 2005). A common empirical approach for dissecting neutral and adaptive variation is to incorporate the genetic structure of neutral markers as a statistical control for testing ecogeographic patterns (Hangartner, Laurila, & Räsänen, 2012; Karkkäinen, Løe, & Ågren, 2004; Kooyers & Olsen, 2012; McKay & Latta, 2002). If one aims to test the effects of a candidate environment on putative loci subject to selection, a specific question is how phenotypic differentiation correlates with environmental gradients and/or fixation indices (such as *F*_st_ or *G*_st_) of neutral genetic markers (e.g., Hangartner et al., 2012; Kooyers & Olsen, 2012). This type of comparison allows us to infer an environmental driver of divergence/convergence in a focal trait (Hangartner et al., 2012). This approach has been taken in plant–herbivore interactions (Agrawal et al., 2015; Sakata et al., 2014), yet whether herbivory is associated with ecogeographic patterns in plant defense remains controversial (reviewed by Han & Maron, 2016).

Plant trichomes (epidermal hairs) have a large extent of variation in their occurrence and density (Ågren & Schemske, 1994; Elle, van Dam, & Hare, 1999; Løe et al., 2007). Binary phenotype of presence/absence of trichomes often follows Mendelian inheritance (Karkkäinen & Ågren, 2002; van Dam, Hare, & Elle, 1999). Genetic studies on natural plant variation have revealed that the heritability of trichome density was at a level greater than 50% (Holeski, Chase‐Alone, & Kelly, 2010; Karkkäinen & Ågren, 2002; Symonds et al., 2005). One major ecological function of trichomes is physical resistance against herbivory (Dalin, Ågren, Björkman, Huttunen, & Kärkkäinen, 2008; Handley, Ekbom, & Ågren, 2005; Hanley, Lamont, Fairbanks, & Rafferty, 2007; Levin, 1973); in addition, trichomes can also protect plants from abiotic stresses such as drought (Gianoli & González‐Teuber, 2005; Sletvold & Ågren, 2012) and frost damage (Agrawal, Conner, & Stinchcombe, 2004). Because of the simple genetic basis and multiple ecological functions, trichome production provides an excellent system to investigate how plant resistance variation is established across heterogeneous landscape involving multiple species interactions and climatic gradients.

**Figure 1:**
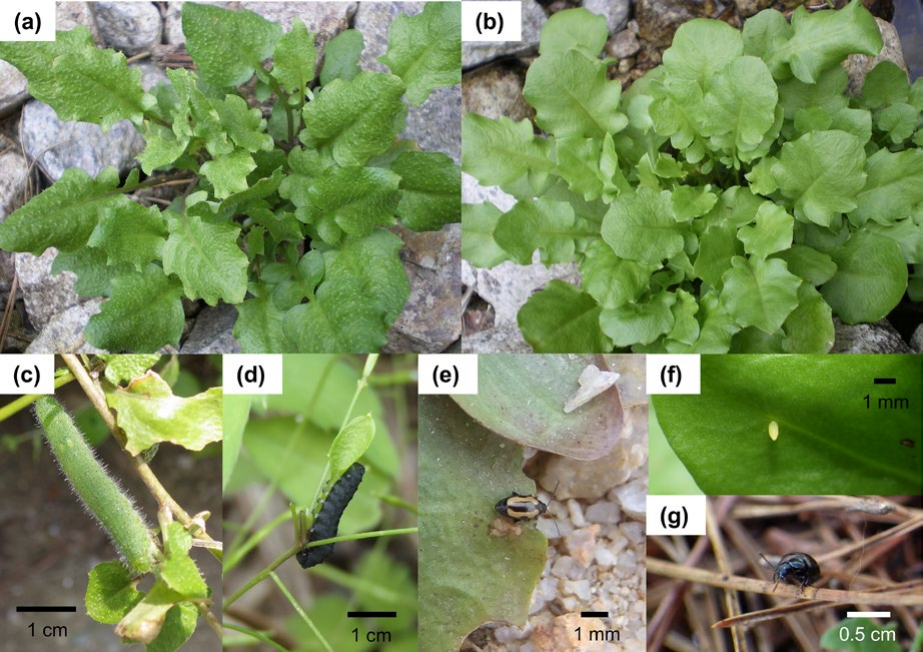
Photographs of *Arabidopsis halleri* subsp. *gemmifera* and insect herbivores: (a) hairy plant, (b) glabrous plant, (c) larva of *Pieris napi*, (d) larva of *Athalia infumata*, (e) adult of *Phyllotreta striolata*, (f) egg of *Pieris napi*, and (g) adult of *Phaedon brassicae*

*Arabidopsis halleri* (L.) O'Kane and Al‐Shehbaz subsp. *gemmifera* (Matsum.) O'Kane & Al‐Shehbaz (Brassicaceae/Cruciferae) have a genetic dimorphism expressed as two morphs, trichome‐producing (hairy: Figure 1a) and trichomeless (glabrous: Figure 1b) plants. As in related species (Bloomer, Juenger, & Symonds, 2012; Hauser, Harr, & Schlötterer, 2001; Kivimäki, Kärkkäinen, Gaudeul, Løe, & Ågren, 2007), the glabrousness of *A. halleri* is associated with mutation of a trichome‐related gene, *GL1* (Kawagoe, Shimizu, Kakutani, & Kudoh, 2011). In this subspecies, it was also discovered that trichome production is costly for plant fecundity (Kawagoe et al., 2011) and growth (Sato & Kudoh, 2016) in the absence of herbivory. Previously, we reported the following body of evidence for antiherbivore functions of trichome production in *A. halleri*: The trichome production could prevent damage caused by *Pieris* butterflies (Figure 1c,f) and the cabbage sawfly *Athalia infumata* (Figure 1d), but it could not defend against the flea beetle *Phyllotreta striolata* (Figure 1e; Sato & Kudoh, 2015). The brassica leaf beetle *Phaedon brassicae* (Figure 1g) mediates negatively frequency‐dependent damage and growth that can promote the maintenance of hairy and glabrous plants (Sato, Kawagoe, Sawada, Hirai, & Kudoh, 2014; Sato & Kudoh, 2016). Thus, we specifically hypothesized that herbivory by butterflies drives divergent selection on trichome dimorphism in relation to their abundance among populations, whereas the brassica leaf beetle particularly stabilizes the dimorphism within a population.

**Figure 2:**
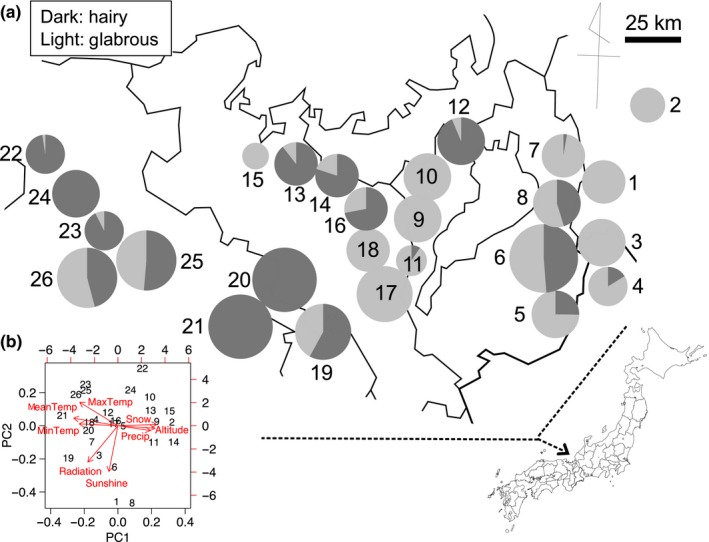
Locality and climatic variation in the present study. (a) Map showing locations of study sites and their frequency of hairy and glabrous plants. The size of pie charts reflects the total number of plants at each site. The site IDs (1–26) correspond to those listed in the Tables S1 and S2. (b) Summary of the principal component analysis (PCA) for eight meteorological variables. Red arrows show contributions of each climatic factor to the first and second principal component, PC1 and PC2

This study aimed to address the following two specific questions. (1) To what extent does leaf damage to *A. halleri* individuals differ among the trichome phenotypes (hairy or glabrous) and study sites? (2) What kinds of ecological factors are responsible for fine‐scale geographic variation in the morph frequency? In this study, we first compared the leaf damage in hairy and glabrous plants among 26 populations in Japan, which were separated from each other by less than 200 km in distance (Figure 2). Then, we evaluated genetic differentiation of a putative trichome locus along gradients of ecological factors, such as herbivory and microclimatic conditions, by incorporating neutral genetic variation as another correlative factor.

## 2 Materials and Methods

### 2.1 Study species

*Arabidopsis halleri* subsp. *gemmifera* is a self‐incompatible, perennial herb distributed across Japan and the Russian Far East (Hoffmann, 2005). In the lowlands of western Japan, plants start flowering in late March or early April and develop clonal rosettes from shoot apical and lateral meristems. A plant with no vegetative connection with others was designated as an individual in this study. Hairy plants produce nonglandular trichomes on their stem and leaf surface, whereas glabrous plants had no trichomes except for those on their leaf margins. The hairy and glabrous phenotypes are associated with allelic variations in an ortholog of *GL1* but not with other genes including two flanking regions (Kawagoe et al., 2011). Wounding did not increase trichome density of hairy *A. halleri* (Sato & Kudoh, 2016), and glucosinolate profiles were not associated with the hairy and glabrous phenotypes in our previous study (Sato et al., 2014). In the field, leaf‐chewing insects on *A. halleri* become abundant in June (Sato & Kudoh, 2015).

### 2.2 Field survey

We surveyed leaf damage and the number of hairy and glabrous plants in 26 populations located within the Kinki area (Figure 2; see also Tables S1 and [Link], [Link]). All the hairy or glabrous plants were counted following a line transect along a continuous habitat of forest edge or pathway. The total number of plants within each site ranged from 40 to 7,400. Leaf damage and herbivore fauna were examined in 2014 and 2015 during early summer (June–mid‐July). We scored visually the proportion of leaf area lost to herbivory as 0 (<12.5%), 0.25 (25% ± 12.5%), 0.5 (50% ± 12.5%), 0.75 (75% ± 12.5%), or 1.0 (>87.5% and <100%) for all leaves in an individual plant. Individual‐level leaf damage was evaluated by averaging the leaf damage scores of all leaves in an individual plant. Leaf damage measurements were taken by a single observer to avoid bias. We also measured the length of the largest leaf (hereafter called maximum leaf length) as it reflects plant's biomass (Sato & Kudoh, 2016). These traits were evaluated for 20–40 hairy or glabrous plants randomly chosen in a population. These plants were sampled at regular intervals within a population; the sampled plants were at least 2 m apart from each other to avoid multiple sampling of a single clone or a spatial autocorrelation of herbivory. All surveys were conducted during a daytime (between 07:30 and 18:00 hr). We identified and counted herbivorous insects observed within the study area in each site. Detailed survey dates and sample sizes are provided in supporting information (Tables S1 and S2).

### 2.3 Meteorological and genetic data

We compiled meteorological conditions for each site using the Mesh Climate 2000 database (Japan Meteorological Agency 2002). This database provides information on annual temperature (°C; mean, maximum, and minimum), solar radiation (MJ/m^2^), sunshine (hr), annual precipitation (mm), and maximum snow cover (cm) at a 1‐km^2^ mesh scale. We added elevation (m) of each site to these variables. These environmental variables were summarized by principal component analysis into two principal components (PC1 and PC2) to avoid multicollinearity. The PC1 and PC2 explained 76% of total variation in the meteorological variables in the summary, and these two components were incorporated into our analysis. The PC1 and PC2 were related to meteorological variables (including temperature and precipitation) and solar‐radiation conditions (including sunshine and radiation), respectively (Figure 2b).

We used the genotype data reported by Sato and Kudoh (2014) to evaluate neutral genetic variation among the studied populations. Sato and Kudoh (2014) analyzed 19 microsatellite loci of 41 *A. halleri* populations sampled across Japan. We used the 26 populations (6–14 plants sampled per site) analyzed in the previous study, of which 25 populations were overlapped with the present study and the data of the nearest population (Fujiwara–Sakamoto) was applied to one site of the current survey (Fujiwara–Ogaito, <1.5 km apart; site 4). Detailed methods and raw genotypic data are available in the previous publication.

### 2.4 Statistical analysis

Data analysis was performed using R version 3.2.0 (R Core Team 2015) and consisted of two steps. First, we conducted type III analysis of variance (ANOVA) to partition sources of variation in the individual plant‐level damage. The response variable was the proportion of leaf area loss in individual plants, which was arcsine‐square‐root‐transformed to improve normality. The explanatory variables were the trichome phenotype, the site ID, study date (*x*‐days from January 1), the study year, and the maximum leaf length. We also applied generalized linear models (GLMs) that dealt with the categorical variable of leaf damage score by using the VGAM package (Yee, 2015: see Appendix S1). Because the results of this GLM are consistent with ANOVA, the ANOVA results are shown to enable straightforward interpretation on the amount of variation explained by each explanatory variable.

Second, we compared the genetic structure of a putative trichome locus and microsatellite loci along the gradients of three ecological factors (population‐level leaf damage, meteorological PC1 and PC2) to infer potential drivers of geographic variation in the frequency of hairy and glabrous plants. We calculated a standardized fixation index, $G_{\mathrm{st}}^{\prime \prime }$ (Meirmans & Hedrick, 2011), for the trichome phenotype and microsatellite data. We estimated $G_{\mathrm{st}}^{\prime \prime }$ of a candidate trichome locus from the morph frequency at each site. The glabrousness of *A. halleri* is a recessive phenotype because the association of glabrous phenotype with a homozygote of glabrous alleles was perfect (Kawagoe et al., 2011). Thus, following Mendelian inheritance, we estimated genotype frequencies as *p *=* *1 − √*f*_g_; *q *= √*f*_g_, where *p* and *q* denote a frequency of hairy and glabrous alleles, respectively, and *f*_g_ is the phenotypic frequency of glabrous plants at a site. We further obtained the expected heterozygosity as *H*_e_ = 2*pq*. After letting *H*_t_ and *H*_s_ be the expected heterozygosity of the entire population and the subpopulation, a fixation index was derived as $G_{\mathrm{st}}^{\prime \prime }$ = *k*(*H*_t_ − *H*_s_)/{(*kH*_t_ − *H*_s_)(1 − *H*_s_)}, where *k* indicates the number of populations sampled (see also eq. 4 in Meirmans & Hedrick, 2011). We calculated *H*_t_ from *H*_e_ of a pooled population and *H*_s_ from the average of *H*_e_ weighted by the number of plants observed in a subpopulation. Population‐level leaf damage was represented by averaging individual‐level damage in a site. We conducted partial Mantel tests in which Pearson's correlations were analyzed with 9,999 permutations among pairwise matrices of the trichome $G_{\mathrm{st}}^{\prime \prime }$, microsatellite $G_{\mathrm{st}}^{\prime \prime }$, and differences of ecological factors between sites. In this analysis, positive or negative correlations between the phenotypic $G_{\mathrm{st}}^{\prime \prime }$ and the environmental differences indicate divergence or convergence of a candidate trait along that focal environmental gradient, respectively. Significant correlations between the phenotypic and neutral fixation statistics indicate the relevance of demographic factors (e.g., gene flow or genetic drift) to the phenotypic variation. We used the “mantel” function (in the ecodist package; Goslee & Urban, 2007) for the Mantel tests and GenoDive version 2.0 (Meirmans & van Tienderen, 2004) for calculation of $G_{\mathrm{st}}^{\prime \prime }$ from the microsatellite data.

Given the recent caution on the partial Mantel test (Guillot & Rousset, 2013), we also applied generalized linear mixed models (GLMMs) to test the effects of leaf damage and climatic components on the morph frequency. The matrix of pairwise population differentiation (microsatellite $G_{\mathrm{st}}^{\prime \prime }$) was used as a random effect in GLMMs. The frequency of glabrous plants in a site was analyzed using a binomial error structure and a “logit link” function. The binary response was an occurrence of a hairy/glabrous plant. We examined the fixed effects of the population‐level damage, meteorological PC1 and PC2 in the GLMMs. Interactions between the damage and meteorological PC1 or PC2 were also included to test climatic dependence of herbivory effects. The “MCMCglmm” package (Hadfield, 2010) in R was utilized to handle an autocorrelation of a random effect in GLMMs. Markov chain Monte Carlo iteration was run 1,000,000 times with a 100,000 burn‐in period and 1,000 thinning interval. Mean and 95% credible intervals were calculated for GLMM parameters after checking mono‐modality of posterior distributions.

## 3 Results

### 3.1 Sources of variation in the individual plant‐level damage

**Figure 3:**
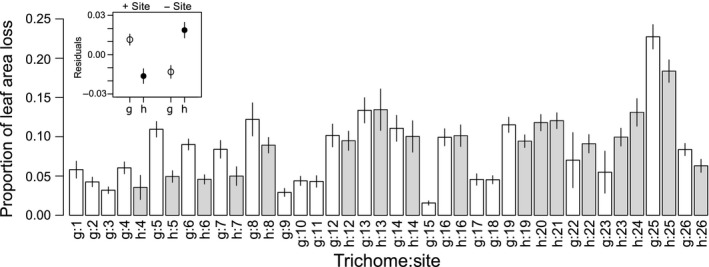
Individual‐level damage (proportion of leaf area loss) of hairy (h; gray bars) and glabrous (g; white bars) plants in 26 populations of *Arabidopsis halleri* subsp. *gemmifera*. Data were pooled between 2014 and 2015. The site IDs (1–26; assigned from east to west) correspond to those in Figure 2 and the Tables S1 and S2. An inset shows differences in residual damage between hairy (h; white) and glabrous (g; black) plants when excluding (‐Site) or including (+Site) the site ID in multiple regressions. Error bars indicate *SE* of mean

The trichome phenotype, study sites, and study dates were significant sources of variation in leaf damage of *A. halleri*, whereas the study years and maximum leaf length did not explain the significant amount of variation in leaf damage (Table 1). The significant negative coefficient of trichome phenotype in the linear multiple regression showed less damage on hairy plants than on glabrous plants (coefficient of hairy phenotype ± *SE* = −0.047 ± 0.009, *t *= −5.086, *p *<* *10^−6^). This decreased damage on hairy plants was visualized by a residual plot standardizing variation among the study sites (inset of Figure 3). However, the considerably larger amount of variation was attributable to the study sites rather than the other sources of variation (Table 1, Figure 3), indicating that among‐site variation was the most significant source of leaf damage. When damages were pooled among study sites, hairy plants were more damaged compared to glabrous plants (inset of Figure 3). The result that among‐site variation was the largest in the individual‐level damage was also supported by our complemental GLM (Appendix S1).

**Table 1:** Analysis of variance table showing the sources of variation in the individual‐level damage

Explanatory	*df*	SS	*F*	*p*
Trichome	**1**	**0.53**	**26.3**	**<10** ^**−6**^
Site	**25**	**7.66**	**15.3**	**<10** ^**−16**^
Year	1	0.01	0.3	.56
Date	**1**	**0.65**	**32.3**	**<10** ^**−7**^
Maximum leaf length	1	0.06	3.2	.07
*Residuals*	1658	33.32	—	—

Degree of freedom (*df*), type III sum of square (SS), *F*‐statistic, and *p*‐value are listed for five explanatory variables. The leaf damage data were arcsine‐square root‐transformed prior to the analysis. Bold values highlight significant sources of variation at *p *<* *.05. Bars indicate no information available.

### 3.2 Ecogeographic differentiation in the morph frequency

**Figure 4:**
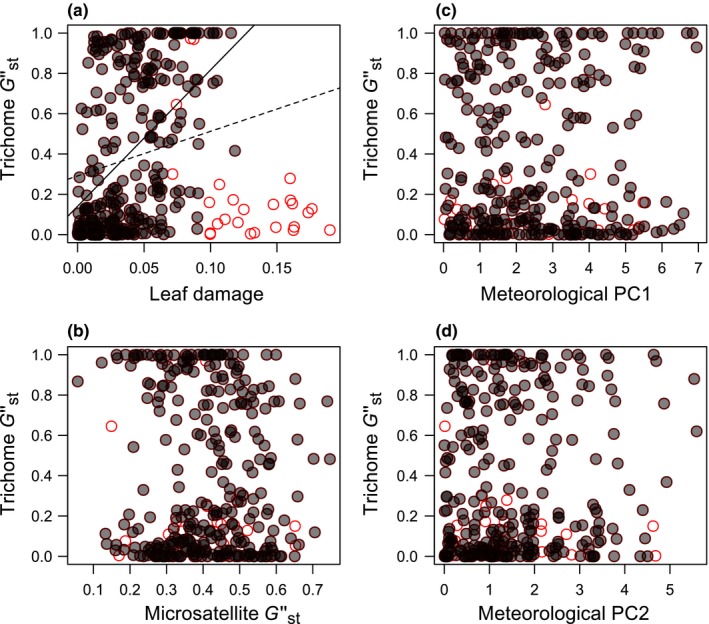
Population differentiation ($G_{\mathrm{st}}^{\prime \prime }$) of a putative trichome locus along the gradient of the population‐level damage (a), neutral genetic differentiation (b), or meteorological components (c, d). The leaf damage and meteorological PCs are shown as differences of a focal variable between sites. Gray closed circles represent a site pair. Red open circles highlight site pairs including the outlier polymorphic population (site 25; Figure 2). Trend lines are added to the entire dataset (dashed line) or to the dataset excluding the outliers (solid line) in panel (a)

Next, we examined ecological and genetic factors responsible for population differentiation in the frequency of hairy and glabrous plants (Table 2). Based on the largest amount of the among‐site variation in the individual‐level damage (Table 1), the population‐level damage was represented as an average leaf damage pooled among the trichome phenotypes and study years. We found a marginally significant tendency in trichome $G_{\mathrm{st}}^{\prime \prime }$ increase along with the increased difference in the population‐level damage (Table 2a, Figure 4a); however, this increase in trichome $G_{\mathrm{st}}^{\prime \prime }$ became significant when site 25 (the site where the brassica leaf beetle *P. brassicae* was especially prevalent) was excluded as an outlier (Table 2b, Figure 4a). These correlations were detected even when the population‐level damage was evaluated using the dataset of glabrous plants only (partial Mantel test with 9,999 permutations, trichome $G_{\mathrm{st}}^{\prime \prime }$ versus damage, *r *=* *.06, *p *=* *.57 for all dataset; *r *=* *.27, *p *=* *.001 for data excluding site 25). The trichome $G_{\mathrm{st}}^{\prime \prime }$ was not significantly correlated with either the microsatellite $G_{\mathrm{st}}^{\prime \prime }$ (Figure 4b) or the meteorological components (Table 2, Figure 4c,d).

**Table 2:** Partial Pearson's correlations (*r*) of the trichome $G_{\mathrm{st}}^{\prime \prime }$ with the population‐level damage, microsatellite $G_{\mathrm{st}}^{\prime \prime }$, and meteorological component matrices

Focal factors	(a) All dataset		(b) Excl. outlier pairs	
	*r*	*p*	*r*	*p*
Population‐level damage	.214	.074	**.487**	**.0001**
Microsatellite $G_{\mathrm{st}}^{\prime \prime }$	−.053	.686	−.035	.658
Meteorological PC1	−.032	.725	−.052	.552
Meteorological PC2	.002	.988	.079	.530

**Figure 5:**
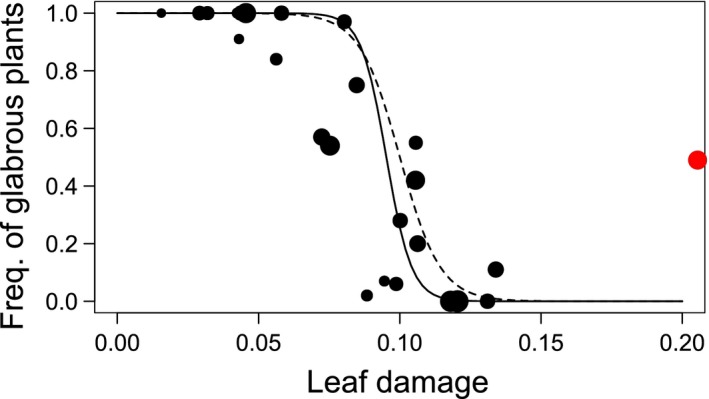
Frequency of glabrous plants plotted against leaf damage at population levels. Size of black circles corresponds to the total number of plants observed within each population. A red circle highlights a polymorphic site where the leaf beetle *Phaedon brassicae* was prevalent (site 25 in Figure 2 and the Tables S1 and S2). Solid and dashed curve indicates the prediction excluding and including the outliner, respectively

Pairwise differences between sites were used for the leaf damage and meteorological PCs. Results of the partial Mantel tests are shown for dataset including/excluding the pairs involving the outlier (red open circles in Figure 5). Bold values highlight statistical significances at *p *<* *.05 with 9,999 permutations.

The complemental GLMM analyses showed a significant negative relationship between the population‐level damage and frequency of glabrous plants (*p *<* *.001: Table 3). The frequency of glabrous plants steeply declined at the point of approximately 10% damage, but the population wherein the leaf beetle *P. brassicae* was prevalent (site 25; 14 adults observed) exhibited an intermediate frequency despite the severest damage of >20% (Table 3, Figure 5). The magnitude of steepness became larger when eliminating the outlier site 25 (solid curve in Figure 5). In contrast, the frequency of glabrous plants was not significantly associated with the temperature‐related component (PC1, *p *>* *.5: Table 3) or the solar‐radiation‐related component (PC2, *p *>* *.15: Table 3). No significant interactions were detected between the leaf damage and the climatic components (*p *>* *.07: Table 3). These GLMM analyses found a negative association between the population‐level damage and frequency of glabrous plants, and further detected the site 25 as an outlier. These results indicate the potential role of herbivory in driving population differentiation in the morph frequency; it is noteworthy that this differentiation becomes weak due to the prevalence of *P. brassicae* at a particular site.

**Table 3:** Effects of population‐level damage, temperature‐related climatic component (PC1), and solar‐radiation‐related climatic component (PC2) on the frequency of glabrous plants within a population

Factor	All dataset				Excl. outlier population			
	Mean	95% LCI	95% UCI	*p*	Mean	95% LCI	95% UCI	*p*
Population‐level damage	−**141.29**	−**248.30**	−**58.30**	**<.001**	−**225.72**	−**327.23**	−**131.50**	**<.001**
Meteorological PC1	0.57	−1.03	2.31	.50	0.24	−0.90	1.52	.70
Meteorological PC2	−0.62	−3.08	1.84	.58	−1.09	−2.81	0.36	.16
Damage × PC1	−15.99	−67.52	28.59	.48	29.56	−3.71	65.35	.08
Damage × PC2	81.00	−2.91	201.78	.07	−21.33	−100.80	44.33	.53

Means with 95% lower and upper credible intervals (LCI and UCI) are shown for the posterior distribution of estimated coefficients in the generalized linear mixed models. Bold values highlight a significant deviation from coefficients of zero at *p *<* *.05.

## 4 Discussion

Geographic variation is widely reported in chemical and physical resistance traits across diverse plant species (e.g., Berenbaum & Zangerl, 1998; Bernhardsson et al., 2013; Løe et al., 2007; Züst et al., 2012). Here, we found major patterns of fine‐scale geographic variation in the frequency of hairy and glabrous *A. halleri*. First, hairy plants were less damaged than glabrous plants within populations, but among‐site variation was largely responsible for the individual‐level damage. Second, the fixation indices revealed that herbivory was a putative driver of frequency differentiation in the trichome dimorphism regardless of neutral genetic differentiation and microclimate conditions. However, the *P. brassicae*‐prevalent site was exceptionally polymorphic so that this outlier could weaken the differentiation of the morph frequency. These findings suggest that the geographic variation in the morph frequency has been shaped jointly by fine‐scale divergence along the herbivory gradients and the prevalence of the brassica leaf beetle in a particular population.

Antiherbivore functions and geographic variation of hairy and glabrous morphs are known in the trichome production of *Arabidopsis* species (Handley et al., 2005; Karkkäinen et al., 2004; Løe et al., 2007; Mauricio, 1998; Sletvold et al., 2010). Mauricio (1998) demonstrated a fitness cost and insect resistance of trichomes in the model species *Arabidopsis thaliana*. The line of studies on *Arabidopsis lyrata* also revealed that trichome production is costly but functions as a resistance against the diamondback moth *Plutella xylostella* (Sletvold et al., 2010), and furthermore, there is adaptive geographic variation associated with herbivory (Karkkäinen et al., 2004; Løe et al., 2007). Specifically, *A. halleri* trichomes act as a resistance trait against *Pieris* butterflies (Sato & Kudoh, 2015), suggesting that the frequently observed herbivore *Pieris napi* could be a selective agent of trichome production in our present survey. Additionally, the trichome production of *A. halleri* is known to be costly for plant growth and fecundity under no herbivory (Kawagoe et al., 2011; Sato & Kudoh, 2016), and thereby, glabrous plants are expected to predominate in the absence of herbivores. Although the population‐level damage was weak (<10%) at many of our study sites, the biomass of glabrous plants was revealed to be 10% greater than that of hairy plants under no herbivory (Sato & Kudoh, 2016). Given the similar levels of herbivory and growth cost for hairy plants, a trade‐off between cost and resistance to herbivory might have led to the divergence in the morph frequency.

Plant trichomes do not always confer resistance to herbivory. For example, trichomes failed to deter herbivory by mirid bugs on Solanaceae species (Hare & Elle, 2002) or by leaf miners on Asteraceae species (Andres & Connor, 2003). In the present survey, we also observed the cabbage sawflies *A. infumata* (Figure 1d) and the flea beetles *P. striolata* (Figure 1e), as well as *Pieris* butterflies (Figure 1c,f). As larvae of *A. infumata* tended to occur less often on hairy plants in our previous field experiment (Sato & Kudoh, 2015), it was possible that the sawflies could be a selective agent on the trichome production. Indeed, hairy plants were less damaged in a polymorphic population where larvae of *A. infumata* were often observed (site 19; Figure 3). In contrast to the sawflies, Sato and Kudoh (2015) could not find any evidence for antiherbivore roles of *A. halleri* trichomes against flea beetles. Consistent with these results, we did not observe less damage on hairy plants compared to that on glabrous plants in natural populations infested with flea beetles (sites 13 and 16; Figure 3). Thus, these small insects were less likely to be a selective agent for trichome production.

In the framework of geographic mosaic of coevolving polymorphism (Thompson, 2005), one specific hypothesis is that among‐population divergent and within‐population balancing selection jointly contribute to geographic variation in plant resistance polymorphism (Berenbaum & Zangerl, 1998; Thompson, 1999, 2005; Zangerl & Berenbaum, 2003). Notably, in *A. halleri*, the brassica leaf beetle *P. brassicae* (Figure 1g) is known to mediate negative frequency dependence in damage and growth between hairy and glabrous plants, which can maintain the trichome dimorphism (Sato & Kudoh, 2016; Sato et al., 2014); however, these balancing effects could not be found in white butterflies and other herbivores (Sato & Kudoh, 2015). The balancing role of leaf beetles provides a plausible explanation for the stasis of the *P. brassicae*‐prevalent polymorphic population despite the severe damage at that site. Moreover, this outlier site weakened the pattern of morph frequency divergence along the gradient of the population‐level damage in our present analysis. Thus, combined with the line of previous works on *A. halleri* trichomes, we hypothesize that mostly divergent and occasionally balancing selection under the multispecific herbivory have jointly contributed to geographic variation in the morph frequency.

Despite the well‐known roles that plant trichomes play in ameliorating the effects of abiotic stresses (Agrawal et al., 2004; Gianoli & González‐Teuber, 2005; Sletvold & Ågren, 2012) and driving population differentiation (Løe et al., 2007; Sletvold & Ågren, 2012; Steets, Takebayashi, Byrnes, & Wolf, 2010), we were unable to identify microclimatic drivers of geographic variation in the frequency of hairy and glabrous *A. halleri*. However, abiotic resistance of trichomes might be detectable at a wider geographic level than the focal scale of our present study. A densely hairy variety of *A. halleri* has been discovered in an exposed semi‐alpine area of central Japan (previously known as *Arabis gemmifera* var. *alpicola*: Ikeda, Setoguchi, & Morinaga, 2010). Kubota et al. (2015) reported a trichome‐related gene other than *GL1* as a candidate of the altitudinal adaptation in *A. halleri*. Although the loss of function of *GL1* and associated glabrousness have been reported in another population of *A. halleri* (Shimizu, 2002), the genetic basis and local adaptation other than the candidate gene *GL1* has yet to be examined along more extreme environments.

One caveat remains about the complex response of *Arabidopsis* trichomes to herbivory. While the presence/absence dimorphism and trichome density are genetically determined (Bloomer et al., 2012; Hauser et al., 2001; Symonds et al., 2005), the trichome production is sometimes induced by wounding (Bloomer, Lloyd, & Symonds, 2014; Holeski et al., 2010; Yoshida, Sano, Wada, Takabayashi, & Okada, 2009). Furthermore, secondary metabolites such as glucosinolates may covary as a correlated defense against herbivory (Mauricio, 1998). Although our previous studies on *A. halleri* failed to discover correlated expressions between the trichome production and glucosinolate profiles (Sato et al., 2014) and increase of the trichome density by mechanical wounding (Sato & Kudoh, 2016), the complex function of trichomes is still possible in *A. halleri*. Another caveat is that the population‐level damage may increase in glabrous‐monomorphic populations as all individual plants are poorly defended in such populations. Given that the ranking of damages to hairy and glabrous plants was opposite within and among populations (inset of Figure 3), our correlative evidence of leaf damage should be carefully interpreted at the individual and population scale.

In summary, the present findings combined with our previous work on *A. halleri* suggest a potential role of multispecific herbivory in shaping the geographic mosaic in the frequency of hairy and glabrous morphs. Evidence has now been accumulated for the local adaption of plants to a particular herbivore regime (Bernhardsson et al., 2013; Sakata et al., 2014; Züst et al., 2012), but if a plant species interacts with multiple herbivore species, this might lead to complex geographic patterns of plant resistance polymorphism via different types of selection at different spatial scales. Further studies at broader scales are required for a comprehensive understanding of the maintenance of genetic variation in plant resistance across a heterogeneous landscape.

## Conflict of Interest

No conflicts of interests concern this study.
