# Supplementary material for: Fine‐scale frequency differentiation along a herbivory gradient in the trichome dimorphism of a wild Arabidopsis
Source: Ecol Evol. 2017 Feb 28;7(7):2133–41. doi: 10.1002/ece3.2830 (PMC5383478; doi:10.1002/ece3.2830)
Supplement: Supplementary file 1 [file ECE3-7-2133-s001.doc]

**Appendix S1.** GLM analysis for sources of deviation in the individual-level damage

As an alternative of ANOVA, we applied a generalized linear model (GLM) that dealt with the categorical response of leaf damage score. The ordered discrete damage (i.e., 0, 0.25, 0.5, 0.75, and 1.0) was fitted by a ‘cumulative’ family and a ‘logit link’ function. The explanatory variables were same as we analyzed using ANOVA. Likelihood ratio tests were applied to GLMs with or without each explanatory variable. The ‘vglm’ function (in the VGAM package: Yee, 2015) was used for this additional GLM. We did not assume complex responses among the five damage categories; namely, the parallel response was selected in the cumulative family.

The trichome phenotype, site ID, study date, and maximum leaf length (a representative of plant size) were significant sources of deviance in the individual-level damage (likelihood ratio test; df = 1, 25, 1, and 1, respectively; *P* < 0.001 for all), but the study year was not a significant source of deviance (deviance = 3.6, df = 1, *P* = 0.06). The amount of deviance explained was 113.6, 11.03 and 196.3 for the trichome phenotype, study date and maximum leaf length, respectively, whereas 3.6 deviance was attributable to the study year. Our GLM analysis showed a significant negative coefficient of the hairy phenotype on the leaf damage score (Coefficient ± SE = –0.301 ± 0.03, *Z* = –10.685, *P* < 10^-16^), indicating less damage on hairy plants than on glabrous plants. However, the considerably larger amount of deviance was explained by the site ID rather than the other four factors (deviance = 1994.7). Thus, these GLMs are consistent with the ANOVA result that among-site variation was the most explanatory source of the individual-level damage.
