# Supplementary material for: Fine‐scale frequency differentiation along a herbivory gradient in the trichome dimorphism of a wild Arabidopsis
Source: Ecol Evol. 2017 Feb 28;7(7):2133–41. doi: 10.1002/ece3.2830 (PMC5383478; doi:10.1002/ece3.2830)
Supplement: Supplementary file 2 [file ECE3-7-2133-s002.doc]

**Table S1** List of 26 populations used in this study. Locality (latitude, longitude, and altitude) and the number of hairy and glabrous plants of *Arabidopsis halleri* subsp. *gemmifera* and herbivorous insects observed are shown for each site.

| ID | Name | Latitude | Longitude | Altitude(m) | #Hairy | #Glabrous | Herbivores observed |
| --- | --- | --- | --- | --- | --- | --- | --- |
| 1 | Sofudani, Gifu | 35°19'N | 136°27'E | 190 | 0 | 488 | 1 larval *Athalia infumata* |
| 2 | Midoridani, Gifu | 35°37'N | 136°36'E | 260 | 0 | 81 | 2 adult *Pieris napi* |
| 3 | Fujiwara-Mikuni, Mie | 35°13'N | 136°27'E | 240 | 0 | 296 | 2 adult *P. napi* |
| 4 | Fujiwara-Ogaito, Mie | 35°10'N | 136°28'E | 120 | 26 | 135 | 2 adult *P. napi*, 3 adult *Phyllotreta striolata* |
| 5 | Kiwada, Shiga | 35°06'N | 136°22'E | 310 | 185 | 548 | 1 adult *P. napi*, 3 larval *A. infumata* |
| 6 | Ojigahata, Shiga | 35°13'N | 136°23'E | 310 | 449 | 602 | 8 adult *P. napi*, 1 larval *A. infumata* |
| 7 | Ibuki, Shiga | 35°24'N | 136°23'E | 350 | 11 | 403 | 1 adult and 1 egg of *P. napi* |
| 8 | Gongendani, Shiga | 35°15'N | 136°22'E | 380 | 134 | 162 | 9 adult *P. napi* |
| 9 | Asibidani, Shiga | 35°13'N | 135°51'E | 410 | 0 | 376 | 2 adult *P. napi* |
| 10 | Umenoki, Shiga | 35°16'N | 135°52'E | 430 | 0 | 511 | 1 adult *P. striolata* |
| 11 | Katsuragawa-Sakashita, Shiga | 35°11'N | 135°51'E | 480 | 6 | 58 | 1 adult *P. napi* |
| 12 | Kutsuki, Shiga | 35°22'N | 135°55'E | 180 | 294 | 20 | *Leaf holes observed for many plants* |
| 13 | Hanase-Yamasu, Kyoto | 35°13'N | 135°47'E | 410 | 656 | 77 | 5 larval *A. infumata*, 7 adult *P. striolata*, 1 *Eurydema rugosa* |
| 14 | Hanase-Bessho, Kyoto | 35°11'N | 135°47'E | 500 | 742 | 185 | 10 larval *A. infumata*, 1 adult *P. striolata* |
| 15 | Miyama, Kyoto | 35°18'N | 135°42'E | 350 | 0 | 46 | *Leaf holes observed but herbivores could not be found* |
| 16 | Kurama, Kyoto | 35°08'N | 135°47'E | 420 | 363 | 141 | 5 adult *P. striolata* |
| 17 | Ohara, Kyoto | 35°10'N | 135°51'E | 330 | 0 | 3686 | *Leaf holes observed but herbivores could not be found* |
| 18 | Shizuhara, Kyoto | 35°07'N | 135°48'E | 230 | 0 | 618 | 1 larval *A. infumata* |
| 19 | Minoh, Osaka | 34°51'N | 135°28'E | 160 | 1353 | 982 | 2 adult *P. napi*, 14 larval *A. infumata*, 6 adult *P. striolata* |
| 20 | Myoken, Osaka | 34°55'N | 135°27'E | 230 | 5292 | 0 | 2 adults and 3 eggs of *P. napi*, 7 adult *P. striolata* |
| 21 | Tada, Osaka | 34°54'N | 135°21'E | 140 | 7335 | 0 | 23 adults and 18 eggs of *P. napi* |
| 22 | Mikohata, Hyogo | 35°15'N | 134°43'E | 320 | 120 | 3 | 7 adults and 6 eggs of *P. napi*, 1 adult *P. striolata* |
| 23 | Takacho-Tada, Hyogo | 35°06'N | 134°53'E | 190 | 75 | 6 | 2 adults and 4 eggs of *P. napi*, 1 *E. rugosa* |
| 24 | Ikuno, Hyogo | 35°10'N | 134°49'E | 360 | 483 | 0 | 3 adults and 13 eggs of *P. napi*, 3 adult *P. striolata,* 2 adult *Phaedon brassicae* |
| 25 | Omoide-gawa, Hyogo | 35°06'N | 134°56'E | 200 | 1051 | 997 | 14 adult *P. brassicae* |
| 26 | Monzen, Hyogo | 35°05'N | 134°54'E | 160 | 1441 | 1699 | 7 adults and 5 eggs of *P. napi,* 1 larval *A. infumata,* 1 adult *P. striolata* |
