# Supplementary material for: Fine‐scale frequency differentiation along a herbivory gradient in the trichome dimorphism of a wild Arabidopsis
Source: Ecol Evol. 2017 Feb 28;7(7):2133–41. doi: 10.1002/ece3.2830 (PMC5383478; doi:10.1002/ece3.2830)
Supplement: Supplementary file 3 [file ECE3-7-2133-s003.doc]

**Table S2** Detailed information on 26 populations used in this study. Shown are dates when hairy and glabrous plants were counted (CountDate) and survey dates (DamageDate) and sample sizes (N_h_ and N_g_) for the population-level damage (AvDamage) in 2014 and 2015.

| ID | Name | CountDate | 2014 | | | | 2015 | | | |
| --- | --- | --- | --- | --- | --- | --- | --- | --- | --- | --- |
|  |  |  | AvDamage | N_h_ | N_g_ | DamageDate | AvDamage | N_h_ | N_g_ | DamageDate |
| 1 | Sofudani, Gifu | 4-May-2014 | 0.040 | 0 | 20 | 12-July-2014 | 0.073 | 0 | 25 | 7-June-2015 |
| 2 | Midoridani, Gifu | 5-May-2014 | 0.033 | 0 | 20 | 13-July-2014 | 0.052 | 0 | 20 | 7-June-2015 |
| 3 | Fujiwara-Mikuni, Mie | 3-May-2014 | 0.029 | 0 | 20 | 12-July-2014 | 0.035 | 0 | 20 | 6-June-2015 |
| 4 | Fujiwara-Ogaito, Mie | 31-Dec-2011 | 0.039 | 0 | 20 | 13-July-2014 | 0.069 | 8 | 20 | 7-June-2015 |
| 5 | Kiwada, Shiga | 3-May-2014 | 0.085 | 15 | 25 | 14-July-2014 | 0.084 | 20 | 25 | 8-June-2015 |
| 6 | Ojigahata, Shiga | 4-May-2014 | 0.068 | 10 | 30 | 12-July-2014 | 0.075 | 30 | 30 | 6-June-2015 |
| 7 | Ibuki, Shiga | 14-June-2014 | 0.111 | 0 | 30 | 14-June-2014 | 0.045 | 6 | 20 | 28-June-2015 |
| 8 | Gongendani, Shiga | 4-May-2014 | 0.099 | 20 | 20 | 26-June-2014 | 0.113 | 20 | 20 | 6-June-2015 |
| 9 | Asibidani, Shiga | 27-Apr-2014 | 0.020 | 0 | 20 | 28-June-2014 | 0.038 | 0 | 20 | 23-June-2015 |
| 10 | Umenoki, Shiga | 12-May-2012 | 0.036 | 0 | 20 | 28-June-2014 | 0.051 | 0 | 20 | 23-June-2015 |
| 11 | Katsuragawa-Sakashita, Shiga | 27-Apr-2014 | 0.046 | 0 | 20 | 28-June-2014 | 0.040 | 0 | 20 | 23-June-2015 |
| 12 | Kutsuki, Shiga | 19-May-2012 | 0.085 | 10 | 20 | 28-June-2014 | 0.109 | 20 | 20 | 23-June-2015 |
| 13 | Hanase-Yamasu, Kyoto | 26-Apr-2014 | 0.142 | 10 | 20 | 21-June-2014 | 0.128 | 20 | 20 | 20-June-2015 |
| 14 | Hanase-Bessho, Kyoto | 26-Apr-2014 | 0.115 | 10 | 20 | 21-June-2014 | 0.100 | 20 | 20 | 20-June-2015 |
| 15 | Miyama, Kyoto | 26-Apr-2014 | 0.021 | 0 | 20 | 21-June-2014 | 0.010 | 0 | 20 | 20-June-2015 |
| 16 | Kurama, Kyoto | 26-Apr-2014 | 0.112 | 7 | 20 | 21-June-2014 | 0.092 | 20 | 20 | 20-June-2015 |
| 17 | Ohara, Kyoto | 26-Apr-2014 | 0.051 | 0 | 31 | 21-June-2014 | 0.040 | 0 | 30 | 20-June-2015 |
| 18 | Shizuhara, Kyoto | 26-Apr-2014 | 0.040 | 0 | 20 | 21-June-2014 | 0.051 | 0 | 20 | 20-June-2015 |
| 19 | Minoh, Osaka | 18-Dec-2011 | 0.095 | 30 | 40 | 18-June-2014 | 0.118 | 30 | 30 | 16-June-2015 |
| 20 | Myoken, Osaka | 10-Dec-2011 | 0.102 | 0 | 40 | 30-June-2014 | 0.134 | 0 | 40 | 12-June-2015 |
| 21 | Tada, Hyogo | 11-May-2014 | 0.093 | 0 | 40 | 30-June-2014 | 0.148 | 0 | 40 | 12-June-2015 |
| 22 | Mikohata, Hyogo | 6-July-2014 | 0.045 | 20 | 3 | 13-June-2014 | 0.131 | 20 | 3 | 13-June-2015 |
| 23 | Takacho-Tada, Hyogo | 6-July-2014 | 0.082 | 20 | 3 | 13-June-2014 | 0.108 | 20 | 2 | 13-June-2015 |
| 24 | Ikuno, Hyogo | 6-July-2014 | 0.122 | 0 | 20 | 13-June-2014 | 0.138 | 0 | 25 | 13-June-2015 |
| 25 | Omoide-gawa, Hyogo | 23-Apr-2014 | 0.250 | 35 | 35 | 3-June-2014 | 0.154 | 30 | 30 | 9-June-2015 |
| 26 | Monzen, Hyogo | 28-Apr-2014 | 0.070 | 15 | 35 | 23-June-2014 | 0.080 | 30 | 30 | 15-June-2015 |
